# Works on Morbid Anatomy

**Published:** 1834-01-01

**Authors:** 


### VIII.

### WORKS ON MORBID ANATOMY.

I. PRINCIPLES AND ILLUSTRATIONS OF MORBID ANATOMY, &c. By *Dr. Hope*, Part VII. October, 1833.

II. ILLUSTRATIONS OF THE ELEMENTARY FORMS OF DISEASE. By *Robert Carswell*, M.D. Professor of Pathological Anatomy in the University of London, &c. &c.

III. ANATOMIE PATHOLOGIQUE DU CORPS HUMAIN. Par *J. Cruveilhier*. Quatorzieme livraison, I et II.

I. THE first of these meritorious works goes on successfully, because ably conducted. The present part opens with the fourth chapter, entitled "Ulceration of the Alimentary Canal." But before giving illustrations of the morbid condition in question, Dr. Hope introduces an exposition of the healthy state of the mucous glands, now so well known as the glands of Peyer, Brunner, &c. In various parts of this Journal, we have given, especially in our review of M. Bretonneau's work, a physiological as well as pathological account of these glands, and therefore we may pass over the subject here, with very little notice. It is sufficient to say that they are very beautifully represented in these plates. Speaking of the isolated and aggregated glands, as more developed in children than in adults, Dr. Hope observes:—

"I am inclined to believe that the high vascularity and irritability prevalent at this age, render the glands subject to temporary and physiological intumescence from healthy stimulants, particularly such as have an aperient tendency; for instance, recent fruits, vegetables, &c.

In confirmation of this view, and as illustrating the superior proneness of the glands to intumescence in children, I may refer to malignant cholera, in which I have almost invariably found the intumescence affect children, while, in adults, it is of comparatively unfrequent occurrence.

Independent of cholera, it is extremely rare, in adults, to see the glands developed to the extent which I have referred to as common in children, unless the patient have died of protracted diarrhœa, or been carried off by an intercurrent disease while convalescent from gastro-enteritis, or fever. In diarrhœa, it is principally the isolated glands that are enlarged; in fever, it is the patches of Peyer. The condition of the glands, in both these cases, is considered by Andral (Path. Anat. ii. 57.) to be that, not of mere intumescence, but of *hypertrophy*, resulting from the irritation with which they had been for some time affected. He believes, therefore, that a great development of the intestinal follicles is not a natural state in the adult. The state, however, when once excited, may, in some instances, continue to exist, without producing any ill effects; as is proved by the fact that it has been found, although the subjects of it had not laboured under any disease of the alimentary canal for a reasonable period before death. In the majority of cases, however, it does produce ill effects: namely, diarrhœa, dysentery, and general irritability of the intestinal canal." 178.

In conclusion, Dr. Hope thinks that he may state, in general, of the mucous glands, that they are more apt to be developed in some individuals than in others—that in children, the liability to intumescence exists in a high degree; whilst in adults, it prevails to so limited an extent as, in most cases, to require for its production, a stimulus amounting to disease.

### The Mucous Glands in Malignant Cholera.

"To the state of the mucous glands in malignant cholera I have already incidentally adverted. As this state is not one of health, and as general authority does not sanction its being classified with the effects of inflammation, it may be appended as a corollary to the present subject, with which it has some affinity from the circumstance, that the physical characters of the isolated glands when enlarged by cholera do not differ from those observed in children in a state of health. Accordingly I have used the same drawing (Fig. 141) to illustrate both. In cholera, it is principally children and young people who present the glandular enlargement. The number of pale, round bodies, like mustard-seeds, is often prodigious; they sometimes pervade every part of the canal, and they are attended by an excessive sero-mucous and albuminous secretion, of which they are no doubt the principal source. During the prevalence of spasmodic cholera in London in 1832, I witnessed numerous cases of this alteration. (For cases, see Description of the Plates, Fig. 141.) Two years previously, it prevailed at a boy's school in Clapham, having originated in the effluvium which escaped on opening a cess-pool. From one of the sufferers, I took the drawing of Fig. 141. Rœderer and Wagler have described and delineated the same, as occurring in an epidemic at Göttingen; two analogous cases are given by Billard (De la Membr. Musq., Obs. 44 and 45), and many others are to be found in the writings of various authors.

The patches of Peyer I have not observed to be enlarged in cholera, except the case had lingered, and terminated with low fever, when their state was found to be that of ordinary inflammation, as described in the following section." 179.

Dr. Hope next proceeds to the consideration of inflammation and enlargement of the mucous glands, representing them in very beautiful and faithful plates. From the description of these plates, we extract the following interesting passage:

"*Case.*—Malignant cholera at Clapham, nearly three years before the general irruption of the same disease in Great Britain. An old cesspool having been opened, and its contents thrown out immediately contiguous to the play-ground of a boys' school, twenty-two boys were, within two days, attacked with the disease.—*Symptoms.* Most alarming vomiting and purging, with prostration appearing, in many, to threaten instant death. Stools for the most part pale, consisting of mucus and muco-purulent matter, slightly streaked with scarlet blood. Matter vomited was, in the great majority of cases, colourless and inodorous. P., in the early stages of collapse, was very frequent, but scarcely perceptible. Skin cold and clammy. In a few, it was, for a short time, hot, with flushing in the face. In some, slight tenderness and tension of the abdomen existed, but no pain was complained of beyond griping before the stools. Twitching, rather than cramp, of the muscles of the upper extremities. The stage of collapse was, in the most favourable cases, succeeded, under the use of stimulants, by a stage of warmth, gentle moisture, and general re-action. My friend Dr. P. M. Latham, (who, with Dr. Chambers and Mr. Pearson, was in attendance,) on seeing thirty children affected with the epidemic cholera in February, 1832, under my care at the St. Marylebone Infirmary, stated that, 'from the identity of the symptoms, he could have imagined himself to be again in the midst of the scene at the boys' school.'

*Treatment.*—Brandy and other stimulants with opium during collapse. Leeches to the heads of a few during re-action. Mustard poultices on the abdomen. Enemata; afterwards, full doses of calomel and opium.

Two died within twenty-five hours; the rest recovered in the course of a week.

*Sectio* of one.—Exterior of the viscera apparently natural. Stomach healthy. Duodenum as represented in Fig. 141, (the specimen being obligingly given to me by Dr. Chambers.) Jejunum exhibited few isolated glands at its upper part, but more below; while the ileum was universally studded with them; and also with patches of Peyer, somewhat enlarged. No glands were found in the great intestine; but its mucous membrane was, throughout, *uniformly* congested, pulpy, and very easily separable from the subjacent tissue. (In the other fatal case which was examined, the isolated glands of the ascending and transverse colon were universally enlarged, giving the whole interior an appearance of tuberculation.) Contents of the bowels were nearly colourless, and had no fæculent or any other peculiar odour.

*Remarks.*—In the Med. Gaz. March 1832, I have given the dissections of one child that died, out of the thirty-one cases in the St. Marylebone Infirmary, and also of some others. The identity of the symptoms and *post-mortem* appearances with those of the present case, proves, if other proof were wanting, that the malignant cholera existed in England before 1832, and was not necessarily an importation from India." lii.

The plates in this part or fasciculus, are equally as well executed as in any of the preceding parts, and on those we have often expended a well-deserved panegyric. Drs. Hope and Carswell will place England on a par at least, with the foremost of our continental neighbours, as far as respects pictorial delineations of diseased structures. They therefore deserve, in an unusual degree, the patronage of the profession in this country. Shame on the man, who can afford a few pounds annually, and who shall withhold his mite of encouragement from such meritorious undertakings!

---

II. The third Fasciculus of the able work of Dr. Carswell is occupied with the continuation of the subject of carcinoma. An account of the second Fasciculus is contained in the thirty-seventh number of this Journal. The conclusion of that part was occupied with the history of the origin of carcinoma. The present is devoted to more tangible facts—to the physical history of the malady.

### Physical Characters of Carcinoma.

1*st Form.* This exhibits considerable varieties. Carcinoma, in the first instance, assumes the particular form of the organ in which it is deposited. At a subsequent period its form is more defined, in proportion as the original structure disappears. Its varieties then are determined by accidental circumstances, and arranged by Dr. Carswell into the *Tuberiform*, *Stratiform*, and *Ramiform*.

The *Tuberiform* arrangement is met with most frequently, and presents great variety. When deposited in organs possessing an uniform density, and submitted to an equal amount of pressure on all sides, it assumes a globular figure. On natural and accidental serous surfaces, though globular at first, it often becomes pyriform afterwards. It assumes a fungiform shape when placed in circumstances which facilitate its lateral or retard its anterior development, as when it meets with a dense unyielding substance during its progress, or, having pierced the skin, is subjected to pressure. When accumulated in separate portions of the cellular tissue into rounded masses, grouped together, and included within a common capsule, it generally presents a lobulated appearance; and in the submucous tissue in particular, it frequently exhibits the external arrangement of the cauliflower or mulberry. That appearance of Carcinoma which resembles the structure of the pancreas, depends generally on the agglomeration of very small globular or pyriform tumours, separated from one another by cellular or cellulo-fibrous tissue, but inclosed in a common capsule.

Such are the physical reasons adduced by Dr. Carswell, for the many tuberiform varieties of carcinoma. Whether those reasons are satisfactory in all instances, it happily does not fall to our province to decide. Yet we may probably be permitted to remark, that not only the configuration, but the actual nature of these morbid growths, now ranked under the generic term of carcinoma, would appear to present some mysterious differences. It has long been noticed that the tumour resembling the structure of the pancreas is not so malignant as that which presents a decidedly medullary appearance; and some of the lobulated tumors of the breast would seem to be merely on the confines of cancer.

The *Stratiform* carcinoma must necessarily be described in the words of Dr. Carswell. It is chiefly seen in the subserous cellular tissue.

"Although it may be deposited in layers of various extent which present no definite arrangement, it more frequently assumes the form of thin circular patches, varying from the breadth of a pin's head to an inch or more in diameter, and presenting an appearance similar to what might be imagined to follow the infusion of a small quantity of milk into a number of isolated points of the subserous cellular tissue. Patches of this kind, which are composed of a substance having the colour and consistence of cream or milk, are most frequently met with beneath the pia mater and pleura pulmonalis, and are remarkably conspicuous in the latter situation on account of their white pearly aspect contrasting so strongly with the surrounding dark colour of the lungs. These patches may occur in the situations I have named without the substance of the brain or lungs presenting any trace of Carcinoma; but I have never met with them unless when the disease existed in some other organ, as the breast, eye, liver, stomach, kidney, or uterus. In some cases, lymphatics filled with fluid carcinomatous matter are observed to communicate with the patches; in other cases no such vessels are observed."

The *Ramiform* arrangement depends upon the presence of carcinomatous matter in the veins. It is witnessed more conspicuously in the kidneys than in any other organ. The whole of its venous system, to the termination of the emulgent vein in the vena cava, is sometimes found distended with carcinomatous matter, either fluid, of cerebral consistence, or possessing the firmness of pancreas. A similar arrangement is sometimes remarkably conspicuous in the stomach, and the abdominal division of the vena portæ is said by Dr. Carswell to furnish a remarkable example also, unconnected with any organ affected with the disease.

Our author observes that another variety of form may be noticed—that dependent on the presence of carcinomatous matter in the lacteals and lymphatics. The former contain it more frequently, perhaps, than the latter. He thinks it incorrect to conclude that lymphatic glands situated in the neighbourhood of an organ affected with carcinoma, always become implicated through the medium of absorption. He remarks that these glands are frequently found to be extensively diseased, the lymphatics presenting no trace of carcinomatous matter. But we apprehend that this test is fallacious, and a common and striking analogical instance will serve to display the objection that readily presents itself to the mind. The syphilitic bubo is seldom attended with any appreciable affection of the lymphatic that connects the gland with the sore.

2. *Bulk.* This varies with the seat of carcinoma, and the pressure to which the tumor is subjected. Perhaps it is never so great when the disease is in the molecular structure of organs, as when it is found upon their free surface. The influence of pressure is conspicuously observed in several striking examples. The carcinomatous tumor of the eye, which for several months may have struggled to reach its external surface, increases with frightful rapidity after destruction of the cornea, and returns with surprising celerity after it has been once removed by operation. The same law will probably account for the speedy augmentation of bulk which a fungous tumor displays, when sloughing or ulceration of the skin has allowed it to protrude externally. Dr. Carswell states that the restraining influence of pressure is more apparent in scirrhoma than in cephaloma, or, to use the more current language of the day, in scirrhus than in fungus hæmatodes. The professional public will probably recollect the confident promises and the failure of Mr. Young, who hoped to cure cancer by systematic pressure. There cannot be a question that he frequently retarded or arrested the growth of the tumor submitted to his bandages, but the patients died, as might safely have been anticipated, from scirrhous contaminations of other parts.

3. *Colour.* An accurate acquaintance with this is essential, because it is a grand characteristic of the disease.

"It is most frequently white, with a shade of grey or blue; sometimes it inclines to yellow, brown, or red, in consequence of the colour of the organ affected with the disease, of the presence of blood, bile, pus, or other fluids in various proportions, or of some other accidental circumstance. But the principal modifications of colour of Carcinoma are seen in the several varieties of both species of the disease; these varieties, as I have already stated, resembling more or less in colour that of the organ or tissue whence have been derived their respective appellations, as that of cartilage, of the pancreas, of fresh or boiled pork, of coagulated albumen or fibrine, of the mammary glands, of the cerebral substance, or a mixture of the latter with blood."

We cannot but remark that however peculiar the tint of carcinoma may appear, it is frequently difficult to distinguish the real nature of the tumor by this very obvious character. Many of the tumors removed by operation from the female breast, have the colour of carcinoma in an eminent degree, when circumstances render it unlikely that they participate in its other and more formidable features.

4. *Consistence.* Dr. Carswell exposes the falsity of the hypothesis, that the soft carcinoma was indurated scirrhus in its origin. The variety of consistence is referred by him to the three following and leading circumstances; —1, The nature of the containing organ—2, The elementary composition of the deposit—and, 3, The changes which occur in the deposit, or the tissues with which it is in contact.

With regard to the first it may be roughly stated, that the softer tissues present the softer varieties of carcinoma, and that pressure tends to effect its consolidation. But the second consideration informs us that this general statement is not universally correct, and that carcinoma, of every consistence, *may* be formed in any and in every organ. Before he adverts, in a particular manner, to the changes that occur in carcinoma and the tissues that surround it, Dr. Carswell describes the chemical and anatomical characters of the deposite.

*Chemical Characters.* Unhappily, the analyses hitherto conducted may be said to display the chemical composition of organs or tissues affected with carcinoma, rather than that of the several varieties of the morbid production itself. The most recent statement is that published by Lobstein, in his "Traité d'Anatomie Pathologique."

Seventy-two grains of scirrhous breast were found to contain—

| | | |
|---|---|---|
| Albumen | 2 | grains. |
| Gelatine | 20 | ,, |
| Fibrine | 20 | ,, |
| Fluid fatty matter | 10 | ,, |
| Water | 20 | ,, |
| | 72 | |

Seventy grains of the uterus in a state of scirrhus contained—

| | | |
|---|---|---|
| Gelatine | 15 | grains. |
| Fibrine | 10 | ,, |
| Fatty matter | 10 | ,, |
| Water | 35 | ,, |
| | 70 | |

According to the same author, it would seem that the chemical composition of cephaloma differs with the different periods of its development. In the first stage, it has been found to contain more gelatine than albumen, and, when in the second stage it has softened, to shew more albumen than gelatine. It requires no laboured criticism to demonstrate, that such chemical analyses are highly unsatisfactory, if not incorrect.

*Anatomical Characters.* The more obvious and gross anatomical characters have already been considered. Dr. Carswell proceeds to those of a more refined description. When a mass of carcinoma is carefully examined, it is found to consist of various proportions of the following elements:—Bloodvessels—carcinomatous matter—and cellular, fibrous, and serous tissues.

The *bloodvessels* vary greatly in number, and sometimes considerably in bulk.

"They are rarely perceptible in any of the varieties of Scirrhoma; are generally few in number in the first and second varieties of Cephaloma (the Organized and Mammary Sarcoma of Abernethy): but in the Medullary Sarcoma they are often so numerous as to form the greater portion of the brain-like tumour in which they ramify. When these vessels are examined in Cephaloma, they are found to vary in diameter from the breadth of a hair to a line, and present that peculiarity of distribution always more or less conspicuous in newly-formed bloodvessels, that is to say, the ramifications of which they are composed communicate with a common trunk at its opposite extremities in the same manner as the hepatic and abdominal divisions of the vena portæ do with the trunk of this vessel. They are frequently varicose; their walls are remarkably delicate; and they have altogether much more a venous than arterial character. They appear to be formed apart from the vascular system of the surrounding tissues, as they can be seen to originate in small red points situated at the centre or at the circumference of the carcinomatous mass, which, at first, assume the appearance of striæ or slender streaks of blood, and afterwards acquire a cylindrical arrangement and ramiform distribution, thereby constituting what may be denominated the *proper circulation* of Cephaloma. The communication which exists between these vessels and those of the organ in which the cephalomatous substance is contained, is frequently very imperfect,—a circumstance which, together with the delicacy of their structure, renders them extremely liable to congestion and rupture. The most minute divisions of these vessels terminate by penicillated extremities in the carcinomatous matter, where they communicate with veins and arteries belonging to the affected organ. The latter vessels, which may be said to form the *collateral circulation* of Cephaloma, are seldom so conspicuous as the former, but there are cases in which they appear to constitute the greater part of the vascular structure of the disease. They proceed in a radiating direction from the pedunculated attachment of a tumour, for example, or arise along its circumference in the cellular tissue which separates it from the neighbouring parts. It is by means of these vessels that the materials required for the nutrition and growth of such tumours are supplied; and, as we shall see afterwards, the partial or even the complete destruction of these and other tumours similary situated, is occasioned by causes which interrupt this their collateral circulation.

The bloodvessels which are seen in Scirrhoma appear to be no other than branches of those which belong to the neighbouring tissues, and which have become enclosed within the substance of which the several varieties of this species of Carcinoma are composed."

This account of the bloodvessels, if strictly accurate, is very good.

Of the *carcinomatous* matter we need scarcely speak.

The *cellular tissue* is frequently small in quantity, and sometimes so fine, as not to be perceptible till after the carcinomatous matter has been separated by pressure and by maceration. It serves the same purpose as the cellular tissue of parenchymatous organs.

The *fibrous tissue* is seldom met with. The *serous tissue* is frequently present, and may form a capsule to the carcinoma, or give rise to cysts of various sizes, containing various fluids.

*Physiological Characters of Carcinoma.* Dr. Carswell remarks, that the vascular organization of the mass is a subject of great importance. On the actual condition of the circulation in the tumour, depends the variety of tint that it displays, and the disposition to bleed that it evinces. If obstruction of the collateral circulation occurs, the congestion that ensues may give rise to internal or external hæmorrhage, or even to the death and sloughing of the mass. The frequency of internal hæmorrhage is attested by the collections of extravasated blood observed in medullary tumours. External hæmorrhage is constantly witnessed, when the tumor protrudes from the surface, and after ulceration of the integument has taken place. The whole of a carcinomatous tumour will sometimes slough away; and although this must be viewed as a rare occurrence, partial mortification is far from uncommon. When the former and less frequent event has been remarked, delusive expectations have been felt that a natural cure was effected. Dr. Carswell states that this general sloughing of the morbid growth has sometimes resulted from the following circumstance. The tumor originally formed beneath the fascia has penetrated through it by a narrow opening, and afterwards increased with great rapidity when released from the pressure that confined it. But the narrow aperture remaining, the isthmus of the tumour has been subject to constriction, and the vascular supply has proved inadequate to the support of the external mass.

Dr. Carswell next considers the process of softening, which he seems to attribute to irritation, congestion, and modification of nutrition. We need scarcely say that this array of medicated terms explains nothing more than that softening occurs. The immediate cause of this effect is involved in the obscurity that shrouds all ultimate facts, and men of a philosophical character will hardly attempt to conceal their ignorance beneath the jargon of a technical phraseology. The efficient cause we have said is obscure—indeed it is totally unknown. But some concomitants occur which are readily detected by the powers of our senses. At the time that the softening is witnessed, the tumor has usually attained some size, and a greater activity is noticed in the vascular organization of the part. In the tubera of the liver, the softening is first effected in the centre, and congested and ramifying vessels are seen there, whilst little appearance of vascular action is displayed at the circumference. A little consideration informs us that the central portion was the original deposite, and has, consequently, existed for the longest time. The healthy tissues of the living body have a long, but a definite period of existence. The morbid growths run a shorter course, but seem to be subjected to a similar law. They display in a rapid series their phases of increase, maturation, and decay, and the latter is effected by the destructive processes of sloughing and of ulceration. A reflection of this nature may serve to diminish our surprise at the course of carcinoma, but the sensible reader will immediately perceive that, though it may tend to account for the *why*, it affords us no insight into the *how*.

The everted edges of the cancerous sore are ingeniously accounted for by Dr. Carswell. He remarks that this their peculiarity of form is produced by the subsequent development of the carcinomatous substance situated beneath them, which, being entirely freed from pressure all round their internal margin, necessarily projects forwards, as it grows, towards the centre of the tumour hollowed out by the softening and sloughing process, and, consequently, carries them gradually upwards and backwards. They acquire, at the same time, a great accession of bulk, and form a rounded undulating border, beneath which the skin is found doubled upon itself, encircling the carcinomatous excavation.

Nerves have never been detected as a new formation in any of the varieties of carcinoma. But the nervous tissue may originate the malady, in common with all the other textures of the body. We lately witnessed at St. George's Hospital a medullary tumour, decidedly originating in one of the nerves of the arm, we believe the external cutaneous. The nerve entered the tumor at its upper end, and again emerged at its lower. In the section of the tumor, no appearance of the nerve displayed itself.

Dr. Carswell considers the development of the subcutaneous venous system, observed in carcinomatous tumors, situated immediately beneath the integuments, as resulting merely from mechanical obstruction to subcutaneous circulation.

We have now presented the substance of the letter-press of this fasciculus.

We have found in it less to criticize than in either of the former, and we think that we observe in Dr. Carswell a more rigorous caution in the admission of what is not strictly induction. There is no excuse for theoretical disquisition in a branch of science so precise as morbid anatomy should be. Where certainty ends, the morbid anatomist should be cautious in proceeding.

The fasciculus before us contains four plates, distinguished for their beauty, and not deficient in exactness. As a work of art, the delineations of Cruveilhier and of Lobstein sink into daubs by its side, and look like the attempt of a pot-house sign to rival a head of Titian or Correggio.

In the first plate are represented the several varieties of scirrhoma—scirrhus, pancreatic sarcoma, lardaceous tissue, and the gelatiniform cancer. The second plate is devoted to the delineation of cephaloma, and comprises the common vascular or organized sarcoma, medullary sarcoma, and fungus hæmatodes. The third plate represents the seat, and some of the more remarkable forms of carcinoma; and the fourth contains examples of the malady as witnessed in the bones.

We cannot conclude without cordially recommending the work to our readers, to the lovers of science, and the patrons of art.

---

III. The two fasciculi of the work of Cruveilhier are devoted to the elucidation of the epidemic cholera. It might seem to many, that the publications on this subject already in existence were more than sufficient to satisfy the world, that the labours and the ingenuity of medical observers had been already expended in vain. But the objects and the character of these fasciculi differ, in every essential particular, from those of the monographs that fill and darken the literary sky. *They* are chiefly occupied with the observation of symptoms, the results of empirical experiments with remedies, and with keen discussions on the question of contagion. The morbid anatomy of this singular disease has been slightly and unsatisfactorily investigated, and that approximation to exactness which constitutes the basis of the treatment of other maladies, is defective in the instances of this very serious one.

Perhaps it may be found that the high tone adopted by Governments and their agents, in asserting the virulent contagiousness of cholera, was unfavourable to a calm and scientific enquiry. The reason of mankind resents such insults; and though subjects may be forced by power to obey, they cannot be compelled to believe. At the present period, pretensions to the right of dictating opinion are particularly ill-timed, and the efforts of legislative and corporate institutions to thrust their views on the body of society have been met by a re-action, perhaps of too violent a character, and by opposition, in which moderation and philosophic truth have been sacrificed at the altar of excited feelings.

The edge of dispute has been blunted, and the acrimony of party has been softened by time, if it has not been mildly subdued by experience. We are now in a condition more adapted to examine closely into facts, and to form unbiassed conclusions. But the swell of the tempest is still upon us, and the minds of the profession are not yet recovered from the intoxicating influence of passion and of prejudice. In the recent works that have been published upon cholera, and in public discussions that have occupied attention, the cautious observer may detect a disposition to extreme opinions, a readiness to indulge in extraordinary hypotheses, and an unphilosophic leaning to empiricism, which evince that the ordinary methods of inquiry are not universally restored.

The work of Cruveilhier to which we now proceed, is eminently calculated to improve our understanding of the malady. It consists of details of individual cases, and of obvious deductions from specific facts. The symptoms during life, and the morbid changes detected in the corpse, are carefully collated, and an accurate history of the structural alterations observed from the earliest to the latest fatal period is thus presented to the reader. This mode of investigation, advantageous as it has proved in all other diseases, is almost exclusively adopted by Cruveilhier in this.

Our author remarks that the similarity of phenomena between the cholera that appeared in Hindostan and that which is now epidemic in Europe, is amply sufficient to establish their identity. He also observes, that the same kind of evidence proves a similar identity between this disease and that which is described in the writings of Hippocrates—that which ravaged Europe in 1534—the epidemic cholera of 1669 and 1676, delineated by Sydenham. He concludes that the sporadic autumnal cholera only differs from the epidemic, as sporadic sore throat differs from the epidemic angina. This unpalatable thrust at the exclusive doctrines of some eminent individuals, may possibly incline them to attach less importance to the facts which he adduces. For such is the constitution of our minds, that we readily credit the statements of a supporter, and entertain an involuntary scepticism towards those of an opponent.

M. Cruveilhier considers the disease under three varieties, to which he seems inclined to append a fourth. 1. Dejections of a choleric character and slight cholera—2. Cholera of a medium degree of severity—3. Severe cholera, with asphyxia or without it. The fourth division includes those cases in which the epidemic influence was less distinctly displayed, but in which it appeared to be exerted.

The first fasciculus of those before us is exclusively occupied with the symptoms of these varieties, and with cases illustrative of their features and their treatment. However interesting these may appear, our object is the morbid anatomy of cholera, and we consequently pass them by. The second fasciculus is chiefly devoted to this part of the subject.

M. Cruveilhier commences what he terms the anatomy and pathological physiology of cholera with an observation so sensible, that we cannot omit it. He remarks that the cases he has given are sufficient to decide, that cholera is a disease which differs from all others, and which demands a separate place in our nosologies. There cannot be a question that this truth has been neglected, and probably much of the difficulty, certainly much of the dispute, concerning the nature and the propagation of the malady, has arisen from the inconsiderate employment of the laws of other diseases to explain the phenomena of this.

The first consideration that occupies our author in treating of the lesions of cholera, is the state of the exterior of the body.

If the patient dies during the period of blueness, the colour of the dead differs little from that of the living corpse. It has been said that the resemblance has occasioned some melancholy errors. The muscles maintain a condition of rigidity comparable to that in the bodies of those who have suffered execution. But extraordinary contractions are seldom observed. Once M. Cruveilhier remarked in a patient, who had died during violent cramps, that the fingers remained widely separated from each other, and the upper and lower extremities were semi-flexed. The coldness of the skin is less intense in the corpse than in the living body. Our author imagines that this is, in great measure, due to the absence of the clammy sweat that moistens the surface during the latter stage of the disease. In several subjects, the temperature of the trunk was as great at the end of eighteen hours as it had been during the period of asphyxia. The putrefactive process is slow, as occurs in subjects exhausted of blood. But the decomposition of the alimentary canal is extremely rapid, as it is in all cases of considerable sanguineous congestion of those organs.

### STATE OF THE DIGESTIVE TUBE IN CHOLERA.

The mucous membrane of the mouth, the pharynx, and œsophagus presents a slightly violet colour. The follicular glands of the œsophagus are enlarged.

The free surface of the peritoneum is more dry than natural, and the fluid secreted is remarkably viscous and adhesive. In a number of instances, the external surface of the stomach and intestines presents a violet colour. The peritoneum is frequently the seat of the punctuated injection, as in peritonitis ; the sero-purulent effusion alone is wanting. A cholera patient, at the Hôtel Dieu, was affected with real peritoneal inflammmation.

Our author has often seen the stomach contracted on itself, and the great cul-de-sac completely effaced. In other instances, the contraction was confined to the pyloric portion. In others, the organ had its usual volume. The mucous membrane of the stomach has appeared to our author less frequently and less severely affected than that of the intestines. Often it presented a natural state. In some cases the follicles were greatly enlarged, in the neighbourhood of the pylorus, in that of the œsophagus, or even throughout the extent of the organ. The membrane was sometimes pale—at other times it offered a rosy tint and an uniform injection. In some individuals the redness was punctuated, disposed in patches or in bands, along the free edge of the longitudinal folds. Sometimes circumscribed ecchymoses were discovered. Sometimes the stomach presented the most unequivocal traces of acute inflammation, traces most frequently observed in those who had died in the secondary stages of the malady.

The small intestine was sometimes contracted for a great extent; at others it was narrowed at regular distances. M. Cruveilhier has noticed invagination without a trace of any inflammation.

In a number of those who sank in the blue stage, the intestines were found filled with the choleric liquid, and that where the dejections had not been profuse. When the patient died at a period subsequent to that of re-action, liquids were not discovered, but in lieu of them a yellowish pultaceous matter, which may not be improperly compared with meconium.

The mucous membrane of the small intestine presented very variable characters. Enlargement of the follicles was next to constant. Sometimes the solitary glandules were exclusively affected, at others the aggregated only were implicated. Both orders of glandules seldom presented marks of inflammation. In one case, and in that alone, a considerable number were in a gangrenous condition.

The colour of the mucous membrane presented every possible variety of tint, from that of the rose to a reddish-black. The discolouration was very different from that occasioned by venous congestion. It was seldom uniform; often were found points and patches of ecchymosis. When a portion of intestine was viewed against the light upon a plate of glass, the vascular arborization was as delicate and as complete as it was possible to conceive.

The traces of sanguineous congestion were almost always most pronounced in the neighbourhood of the ileo-cæcal valve, and progressively diminished, as the distance from this point increased. But M. Cruveilhier has arrived at the conclusion that the large intestine is that which presents the most constant and most considerable cadaveric alterations.

The great intestine varied extremely in volume, being sometimes filled and distended by liquids, sometimes contracted at regular distances. In this, at the early period, the rice-water evacuations were chiefly discovered. After re-action had ensued a yellowish or greenish pulp, resembling meconium, was commonly observed.

The mucous membrane displayed many varieties of coloration. Sometimes the tint was natural; sometimes that of the hortensia; sometimes the membrane was injected in an arborescent manner, and displayed points of ecchymosis, large black patches, occasioned by effusion of blood into the cellular tissue external to the mucous, or into the mucous itself. In only one instance did M. Cruveilhier discover a really gangrenous condition of the membrane.

The follicles were frequently much enlarged, especially in the neighbourhood of the ileo-cæcal valve. In a number of cases the follicle was surrounded by a sort of red areola, produced by penicillated injection.

The spleen and the liver offered little worthy of remark. The bladder was contracted in those who died in the period of asphyxia, but contained some urine in those who sank in the stage of re-action.

In alluding to the condition of the heart and of the blood, a condition with which our readers must be well acquainted, the author indulges in an observation on the chemical examinations of the latter, which we fear must be owned to be just. Chemistry, says he, has been vainly invoked, and the different results at which chemists have arrived, admit of a comparison with those which have been obtained by an analysis of atmospheric air, instituted to discover the nature of miasma. The accurate researches of the candid Dr. Stevens will probably occur to the reader's mind. He may possibly reflect on the loud pretensions by which others, as well as that admirable chemist and successful physician, succeeded in attracting the attention of the public, and the admiration of the more credulous of the profession.

The cerebrum and cerebellum were injected with blood, as they are in a state of asphyxia. The pia mater was injected, and sometimes slight ecchymoses were observed. The nervous system of organic life has appeared to M. Cruveilhier perfectly natural, and he feels a difficulty in conceiving how M. Delpech could discover in the semilunar ganglia any marks of inflammation.

The foregoing is an abridged enumeration of the facts disclosed on the examination of the dead. A resumé, or a series of inductive propositions, is appended by the author, and is worthy of attention.

1o. The epidemic cholera cannot be ranked amongst those diseases which entirely admit of explanation by alterations of structure. It is true that in some cases decisive alterations are discovered, but in others they are trifling, dubious, or absent.

2do. It is no less clear that the most remarkable anatomical lesions have their seat in the alimentary canal, and more particularly in the great intestine and the inferior part of the small. These lesions sometimes consist in the development of the solitary and aggregated follicles—sometimes in discoloration, more or less decided—injections—ecchymoses—and sometimes in gangrene of the mucous membrane. But none of these lesions can be looked upon as constant, their existence cannot be determined à priori, and frequently their intensity is inversely in proportion to the symptoms.

3tio. The presence of the cholera fluid in the intestinal canal is the only appearance that M. Cruveilhier can denominate specific. It is constantly observed in those who have sunk in the period of asphyxia.

4to. The blood presents an almost invariable and peculiar character in cholera asphyxia; and the apparatuses of circulation and of respiration presented the characters noticed in asphyxia.

Our author next considers the various explanations or theories of the disease that have been offered to the public. The nature and the limits of this article forbid us to enter on these parts of the subject. We shall venture to indulge in but a few remarks.

When we calmly contemplate the cases presented to our view, the well informed, cautious, and practical physician very readily perceives, that some must be fatal under any plan of treatment, and that many will do well under any kind of remedy. The ignorant, the designing, or the enthusiastic, select the latter class as the subjects of experiment and the evidence of success.

A careful consideration of the facts disclosed by the dissection of the dead, confirms, if it tends to moderate, our knowledge. We perceive that in many instances no lesion is discovered which will fairly explain the severity of symptoms. But we also remark that in a large proportion alterations are detected, that these become more constant in the ratio of the duration of the case, and that if they are not exclusively inflammatory in their character, they demonstrate at all events sanguineous determinations. Of the frequent occurrence and continual risk of such determinations, the practitioner should be aware, and the facts of morbid anatomy will lead him to endeavour to prevent and to remove them.

When symptoms are considered, they are found to correspond, in the majority of instances, with the lesions which are noticed. During the asphyxia period, the gravity of the symptoms might lead us to suspect, what the scalpel of the dissector has enabled us to ascertain—that no prominent, no individual alterations, are sufficient to explain them. Cholera, in this period, resembles those rapid and malignant fevers, the cause of which may exist in the fluids, or in the nervous system, but which has hitherto baffled inquiry, and defied the treatment of reason or of accident. But if medicine, or the powers of the patient's constitution, have enabled him to rally, another train of symptoms is established, more consistent in their characters with those of common occurrence, attended with some marks of inflammatory action, and connected with the lesions to which we have adverted.

When we pass in review the multifarious grades of this singular disease—when we look at the important difference in symptoms, and the marked corresponding difference in lesions, we are forced, however unwillingly, to the conclusion, that one plan of treatment cannot suit all. In the slighter forms, diarrhœa may constitute the only symptom—in the most severe, there is positive asphyxia—in the secondary stages, there is inflammation of the mucous membrane. He who can conceive that this or that remedy is adapted to the whole, must have a very limited experience, or a large capacity of belief.

We do not feel inclined to introduce our own opinions. We may venture, however, to observe, that we have found little difficulty in arresting the cholera diarrhœa by astringents, combined with hydrargyrus c̄ creta and Dover's powder, or calomel and opium, to improve and maintain the secretions. Some practitioners commence with aperient remedies; we have found it better to conclude with them. We think that it is best to check the diarrhœa, and then to restore the functions and secretions.

We cannot but suspect that stimulants present the best chances of success in severe collapse. But the object of the practitioner should be not to push them too far, and to watch for the moment when prudence may allow him to withdraw them. The vomiting will sometimes render it impossible to administer stimulating remedies, indeed to administer any by the mouth. In such cases we have found it better to withhold all medicine, and allow the patient to consult his inclination in drinking of warm or of iced diluents. But often it is possible and very useful to exhibit those remedies or even food by the rectum, which the stomach refuses to receive. The experiments of Dupuytren have demonstrated the powers of opiate enemata. We have frequently administered injections of liquid nourishment with wine, or brandy, and with opium, and have found them decidedly useful in cases of cholera of the worst description.

So soon as the secondary fever is appearing, the practitioner should cautiously endeavour to ascertain if local determinations are occurring. If tenderness exists in the abdomen, or pain or uneasiness in the head, leeches or cupping should be used with discrimination. To this point we would direct particular attention. The secondary fever of cholera differs from the ordinary typhus, only in the inferior powers of the patient, and the greater incapability of supporting reduction. Like typhus, then, it should be treated —by calomel, by mild aperients, by local bleeding or blistering to subdue particular determinations.

Such is a slight sketch of the principles of treatment which we have found most useful. The items of the plan would be out of place here, and perhaps they will suggest themselves to all intelligent practitioners. But setting opinions aside, we intreat the profession to investigate this as they would other maladies; to observe the symptoms of each case; to study the alterations of structure, and to connect the two with each other and with the remedies, and to form a rational conception of the disease, if they cannot discover an universally successful method of treatment. The advantages obtained in this deliberate manner are always certain, if they are not brilliant.

